# The value of dynamic FDG PET/CT in the differential diagnosis of lung cancer and predicting EGFR mutations

**DOI:** 10.1186/s12890-024-02997-9

**Published:** 2024-05-10

**Authors:** Xieraili Wumener, Yarong Zhang, Zihan Zang, Fen Du, Xiaoxing Ye, Maoqun Zhang, Ming Liu, Jiuhui Zhao, Tao Sun, Ying Liang

**Affiliations:** 1 Department of Nuclear Medicine, National Cancer Center/National Clinical Research Center for Cancer/Cancer Hospital & Shenzhen Hospital, Chinese Academy of Medical Sciences and Peking Union Medical College/Shenzhen Clinical Research Center for Cancer, Shenzhen, China; 2 Shenzhen Middle School, Shenzhen, China; 3 Department of pathology, National Cancer Center/National Clinical Research Center for Cancer/Cancer Hospital & Shenzhen Hospital, Chinese Academy of Medical Sciences and Peking Union Medical College/Shenzhen Clinical Research Center for Cancer, Shenzhen, China; 4 Paul C. Lauterbur Research Center for Biomedical Imaging, Shenzhen Institute of Advanced Technology, Chinese Academy of Sciences, Shenzhen, China

**Keywords:** Dynamic imaging, PET/CT, ^18^F-FDG, Non-small cell lung cancer, Epidermal growth factor receptor

## Abstract

**Objectives:**

^18^F-fluorodeoxyglucose (FDG) PET/CT has been widely used for the differential diagnosis of cancer. Semi-quantitative standardized uptake value (SUV) is known to be affected by multiple factors and may make it difficult to differentiate between benign and malignant lesions. It is crucial to find reliable quantitative metabolic parameters to further support the diagnosis. This study aims to evaluate the value of the quantitative metabolic parameters derived from dynamic FDG PET/CT in the differential diagnosis of lung cancer and predicting epidermal growth factor receptor (EGFR) mutation status.

**Methods:**

We included 147 patients with lung lesions to perform FDG PET/CT dynamic plus static imaging with informed consent. Based on the results of the postoperative pathology, the patients were divided into benign/malignant groups, adenocarcinoma (AC)/squamous carcinoma (SCC) groups, and EGFR-positive (EGFR+)/EGFR-negative (EGFR-) groups. Quantitative parameters including K_1_, k_2_, k_3_, and K_i_ of each lesion were obtained by applying the irreversible two-tissue compartmental modeling using an in-house Matlab software. The SUV analysis was performed based on conventional static scan data. Differences in each metabolic parameter among the group were analyzed. Wilcoxon rank-sum test, independent-samples T-test, and receiver-operating characteristic (ROC) analysis were performed to compare the diagnostic effects among the differentiated groups. *P <* 0.05 were considered statistically significant for all statistical tests.

**Results:**

In the malignant group (*N* = 124), the SUV_max_, k_2_, k_3_, and K_i_ were higher than the benign group (*N* = 23), and all had-better performance in the differential diagnosis (*P* < 0.05, respectively). In the AC group (*N* = 88), the SUV_max_, k_3_, and K_i_ were lower than in the SCC group, and such differences were statistically significant (*P* < 0.05, respectively). For ROC analysis, K_i_ with cut-off value of 0.0250 ml/g/min has better diagnostic specificity than SUV_max_ (AUC = 0.999 vs. 0.70). In AC group, 48 patients further underwent EGFR testing. In the EGFR (+) group (*N* = 31), the average K_i_ (0.0279 ± 0.0153 ml/g/min) was lower than EGFR (-) group (*N* = 17, 0.0405 ± 0.0199 ml/g/min), and the difference was significant (*P* < 0.05). However, SUV_max_ and k_3_ did not show such a difference between EGFR (+) and EGFR (-) groups (*P*>0.05, respectively). For ROC analysis, the K_i_ had a cut-off value of 0.0350 ml/g/min when predicting EGFR status, with a sensitivity of 0.710, a specificity of 0.588, and an AUC of 0.674 [0.523–0.802].

**Conclusion:**

Although both techniques were specific, Ki had a greater specificity than SUVmax when the cut-off value was set at 0.0250 ml/g/min for the differential diagnosis of lung cancer. At a cut-off value of 0.0350 ml/g/min, there was a 0.710 sensitivity for EGFR status prediction. If EGFR testing is not available for a patient, dynamic imaging could be a valuable non-invasive screening method.

## Introduction

Lung cancer is one of the most common cancers worldwide and the leading cause of cancer-related deaths [1]. In China, it ranks first with a 30% mortality rate [2]. Early detection, accurate diagnosis, and the development of individualized treatment plans play an important role in improving survival rates.

The non-invasive ^18^F-fluorodeoxyglucose (FDG) positron emission tomography/CT (PET/CT) has been widely used in differential diagnosis, staging, and prognosis assessment of lung cancer. Because FDG is not a tumor-specific imaging agent and the standardized uptake value (SUV), a semi-quantitative metabolic parameter, is affected by a variety of factors (such as scan time, blood glucose level, etc.) [3, 4], differentiating between benign and malignant lesions can be difficult. For example, the differential diagnosis of tumors and some inflammatory lesions (such as granulomatous, tuberculosis, and infectious diseases) poses a challenge. Previous studies have shown that, in regions where endemic tuberculosis is highly prevalent, the specificity of FDG PET/CT in the differential diagnosis of benign and malignant lung diseases is reduced by 16-25% [5–7]. For this reason, it is imperative to enhance the FDG PET/CT’s efficacy in differential diagnosis in areas like China where granulomatous lesions and tuberculosis are more prevalent. Contrast to static SUV scan, dynamic FDG PET/CT (dPET/CT) continuously acquires imaging data over a certain period of time. By reconstructing dynamic images, absolute quantitative metabolic parameters can be computed based on a suitable compartment model. For FDG, net influx rate K_i_, FDG delivery rate K_1_, and phosphorylation rate k_3_ can be obtained based on the two-tissue irreversible compartment model [8]. dPET/CT extracts physiological parameters which can better reveal the pathophysiological mechanisms of diseases. Such quantitative analysis has potential advantages in the differential diagnosis of benign and malignant, thus reflecting tumor characteristics and monitoring treatment response [8–15]. Dynamic metabolic characteristics have been the subject of numerous prior studies in tumor differential diagnosis; nevertheless, there are relatively few studies predicting the pathological type of lung cancer or EGFR mutations.

This prospective study aimed at the diagnostic efficacy of dynamic metabolic parameters (K_1_, k_2_, k_3_, and K_i_) and SUV_max_ in differential diagnosis of lung cancer. In addition, we want to explore the value of each metabolic parameter in predicting the type of lung cancer pathology and EGFR mutations.

## Methods

### Patients and inclusion criteria

The study was approved by the ethics committee of Cancer Hospital & Shenzhen Hospital, Chinese Academy of Medical Sciences (KYLH2022-1). All patients signed a written informed consent according to the Declaration of Helsinki before the FDG PET/CT imaging.

Prospective consecutive enrollment of 191 patients who underwent dPET/CT (65 min, chest) + static FDG PET/CT (sPET/CT, 10–20 min, whole body) scans from May 2021 to April 2023 were included. Inclusion criteria were: (1) lung nodules (short diameter ≥ 0.8 cm) or masses detected by chest CT, (2) no anti-inflammatory or anti-tumor therapy prior to FDG PET/CT scan, and (3) puncture and/or surgical pathology results within two weeks of having an FDG PET/CT scan and had complete pathology data. Exclusion criteria were as: (1) previous history of tumor, (2) multiple nodules or masses in both lungs (≥ 2 foci with a short diameter greater than 0.8 cm) detected by chest CT, (3) pure ground-glass density foci detected by chest CT, (4) not confirmed by puncture and or surgical pathology and, (5) unwilling to cooperate. As a result, 147 patients successfully underwent dPET/CT + sPET/CT scans were included in this study. For each patient, imaging characteristics were collected, including long-diameter primary foci, short-diameter primary foci, and dynamic/static quantitative parameters. In addition, patients’ clinical information was collected, including age, gender, TNM stage [16], pathological type, and EGFR mutation status.

### Data acquisition and reconstruction

All patients fasted for at least 6 hours before scans that performed on the PET/CT scanner (Discovery MI, GE Healthcare, Milwaukee, United States). Blood glucose was maintained to be lower than 8.0 mmol/L. The patient first underwent the chest CT in the supine position with the arm raised. The CT parameters were tube voltage of 120 kV, tube current setting of 10–220 mA, pitch of 1.375:1, and noise index of 20. The PET scans covering the chest region were initiated immediately after the injection of ^18^F-FDG ( 264.8 ± 37 MBq)from an intravenous indwelling needle. A scan lasted for 65 min. Dynamic scan data were then partitioned into 28 frames as follows: 6 × 10 s, 4 × 30 s, 4 × 60 s, 4 × 120 s, and 10 × 300 s. After the dynamic scan, the patients underwent a whole-body CT scan from the head to the mid-femur in a supine position with the arms raised. An additional whole-body sPET scan was then performed. For both PET scans, the attenuation corrections were performed using CT data, and the PET reconstructions were performed using the Block sequential regularized expectation maximization (BSREM) reconstruction algorithm with 25 iterations and 2 subsets.

### PET data analysis

According to kinetic compartmental modelling, a set of linear, first-order differential equations can be used to calculate the rate constants at which the tracer exchanges between the blood and tissue compartments. Based on the two-tissue irreversible compartment model we obtained quantitative parameters, including K_1_, k_2_, k_3_, and K_i_. In this model, unidirectional uptake of ^18^F-FDG was assumed (i.e., k_4_ = 0), with irreversible trapping in tissue as ^18^F-FDG-6-PO4 [17]. The image-derived input function (IDIF) was extracted from the ascending aorta by drawing a 10-mm-diameter ROI on six consecutive slices in an image obtained by combining early time frames (0–60 s), where the effects of motion and partial volume were less prominent than in the left ventricle. Parametric images of each dynamic scan were generated using voxel-based analysis using an in-house MATLAB program (MathWorks, version 2018b) that was similar to the procedure in [18]. The uptake differences in blood and plasma was not accounted for in this study. Given a large number of voxels, the Lawson-Hanson non-negative least squares algorithm was applied to solve a linearized problem instead of the conventional nonlinear one [19]. The 3D volume-of-interest (VOI) of each lesion was delineated using the semi-automatic methods with a threshold of 40% SUV_max_ in ITK-snap software (version 4.9). Then the segmented VOI was applied to the K_1_, k_2_, k_3_, and K_i_ parametric images to extract the quantitative measurements of each scan. For the lesions with surrounding physiological uptake or poorly delineated peripheral vessels, 3D VOI was manually delineated slice-by-slice by two experienced nuclear medicine physicians with more than 10 years of experience. Commercialized software supplied by vendor only can calculate Ki but no other parameters. Similarly, most open-source softwares do not have the capability to conduct full kinetic modelling.

Static images were independently reviewed by the same nuclear medicine physicians. The long and short diameters of the primary lung foci were measured on a CT image with 2.79-mm slice thickness. In case of disagreement between the two raters, the consensus was reached by discussion.

### Pathology diagnosis and mutation detection

All punctured and or postoperative specimens were fixed in formalin, dehydrated, and paraffin-embedded. Four-micron sections of each tissue were stained with hematoxylin and eosin (H&E) and Immunohistochemistry. Immunohistochemical studies for P63, P40, TTF1, CK7, and Napsin-A were performed for all the cases using automatic immunohistochemical staining system (Roche, BenchMark ULTRA). Two experienced pathologists performed the diagnosis independently based on microscopic presentation and immunohistochemical results. If there is disagreement, the diagnosis is clarified after a full departmental discussion. Lung cancers were classified according to the 2021 WHO classification. Analysis of EGFR mutations based on the principle of Amplification refractory mutation system PCR (ARMS-PCR) technique with an AmoyDx EGFR Mutations Detection Kit (ADx-ARMS). The operation process is carried out according to the kit instructions.

### Statistical analysis

Differences in static and dynamic metabolic parameters were compared between benign and malignant groups, adenocarcinoma (AC) and squamous cell carcinoma (SCC) groups, and EGFR positive (EGFR+) and EGFR negative (EGFR -) groups using the Wilcoxon rank-sum test or independent-samples T-test based on whether they follow normal distribution or not. Receiver-operating characteristic (ROC) curves were constructed to obtain the cut-off value of the K_i_ for differential diagnosis and prediction of EGFR status. A *P*-value less than 0.05 was considered statistically significant. All statistical analyses were performed using R statistical software (version 4.1.1).

## Results

### Patient and lesion characteristics

Patient and lesion characteristics are presented in Table 1. Of the 147 patients, the median age was 59.48 years (range, 27–84), and the number of male and female patients was 84 (57.14%) and 63 (42.86%), respectively.

**Table 1:** Characteristics of the patient and lesions

Characteristic	Distribution
**Age** (years), Mean ± SD(range)	59.48 ± 11.60 (27–84)
**Sex**	
Male	84 (57.14%)
Female	63 (42.86%)
**Benign group**	23 (15.65%)
Inflammation	13 (56.52%)
Granuloma	5 (21.74%)
Tuberculosis	4 (17.39%)
pulmonary sequestration	1 (4.35%)
**Malignant group**	124 (84.35%)
Adenocarcinoma	93 (75.00%)
Squamous cell carcinoma	17 (13.71%)
Small cell carcinoma	5 (2.52%)
Primary lymphoepithelioid carcinoma lung	3 (4.03%)
Others	9 (7.26%)
**Stage**	
I/ II/ III / IV	31 (25.00%) / 16 (12.90%) / 29 (23.39%) / 48 (38.71%)
**EGFR detection**	48
EGFR (+)	31 (64.58%)
EGFR exon 18/19/20/21/18 + 20 mutation	2 (4.17%) / 19 (39.58%) / 2 (4.17%) / 7 (14.58%) / 1 (2.08%)
EGFR (-)	17 (35.42%)

Based on pathological results, 23 (15.65%) patients were classified in the benign group 124 (84.35%) patients were classified in the malignant group. The detailed pathological types in the benign and malignant groups are presented in Table 1. Forty-eight of the 93 AC patients underwent EGFR status testing, resulting 31 (64.58%) patients in the EGFR (+) group and 17 (35.42%) patients in the EGFR (-) group.

### FDG PET/CT parameter analysis between benign and malignant groups

Table 2 shows the parameter analysis for both dPET/CT and sPET/CT in benign and malignant groups. In sPET/CT, SUV_max_, long and short diameters showed significant difference between benign and malignant groups (3.20 [1.85;6.50] vs. 9.35 [5.60;13.10], 2.07 (± 1.16) vs. 3.68 (± 1.89) cm, 1.42 (± 0.81) vs. 2.87 (± 1.43) cm, *P* < 0.001, respectively).

**Table 2:** PET/CT parameter analysis of benign and malignant groups

Parameters parameters	Benign group (*N* = 23)	malignant group (*N* = 124)	*P*
Long Diameter (cm)	2.07 (± 1.16)	3.68 (± 1.89)	< 0.001
Short Diameter (cm)	1.42 (± 0.81)	2.87 (± 1.43)	< 0.001
SUV_max_	3.20 [1.85;6.50]	9.35 [5.60;13.10]	< 0.001
K_1_ (ml/g/min)	0.1661 [0.0974;0.3561]	0.1239 [0.0957;0.1910]	0.092
k_2_ (min ^− 1^)	0.4077 [0.3089;0.9022]	0.2494 [0.1418;0.4353]	< 0.001
k_3_ (min ^− 1^)	0.0330 [0.0204;0.0489]	0.0632 [0.0344;0.0888]	0.001
K_i_ (ml/g/min)	0.0102 [0.0069;0.0142]	0.0267 [0.0183;0.0422]	< 0.001

Long and short diameter values was expressed as mean ± standard deviation with independent-samples T-test, metabolic parameters were expressed as median [interquartile spacing) with Wilcoxon rank-sum test

In dPET/CT, the average K_i_ and k_3_ in the benign group (0.0102 [0.0069;0.0142] ml/g/min,0.0330 [0.0204;0.0489] min^− 1^) were lower than those in the malignant group (0.0267 [0.0183;0.0422] ml/g/min,0.0632 [0.0344;0.0888] min^− 1^). All differences were statistically significant (*P* ≤ 0.001, respectively). The k_2_ in the benign group (0.4077 [0.3089;0.9022] min^− 1^) was higher than those in the malignant group (0.2494 [0.1418;0.4353] min^− 1^) with statistical significance (*P* < 0.001). However, the K_1_ did not show significant differences between the benign and malignant groups (0.1661 [0.0974;0.3561] vs. 0.1239 [0.0957;0.1910] ml/g/min, *P* = 0.092).

### ROC analysis and cut-off values of FDG PET/CT metabolic parameters

**Fig. 1:**
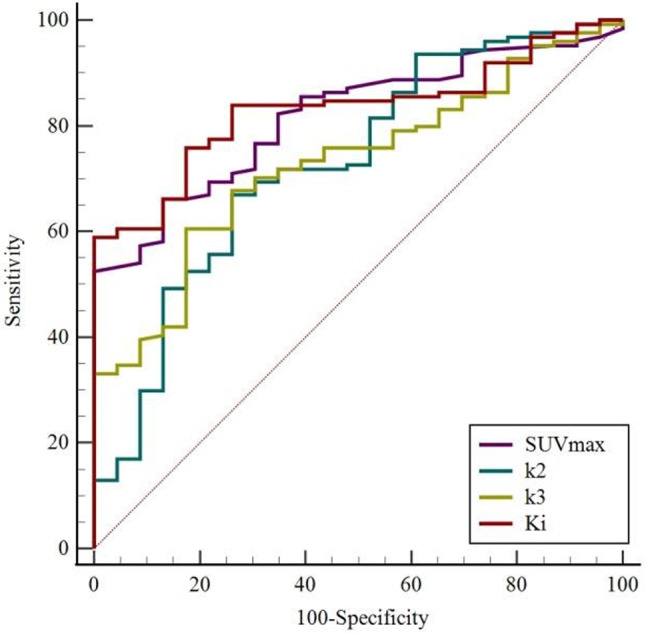
The ROC curves showed parameters for the differential diagnosis of benign and malignant groups

Based on the results of the last section, the metabolic parameters SUV_max_, k_2_, k_3_, and K_i_ entered into the ROC analysis. As shown by the ROC curves (Fig. 1), the cut-off value of SUV_max_ was 7.45, with an AUC of 0.819 (0.743–0.895), a sensitivity of 0.661, and a specificity of 0.870.

For the dynamic parameters (Table 3), the cut-off value of k_2_, k_3_, and K_i_ were 0.338 min^− 1^ (AUC 0.729 [0.614–0.845], sensitivity 0.669, specificity 0.739), 0.053 min^− 1^ (AUC 0.728 [0.631–0.826], sensitivity 0.605, specificity 0.826), and 0.025 ml/g/min (AUC 0.830 [0.761–0.900], sensitivity 0.589, specificity 0.999), respectively.

**Table 3:** Diagnostic efficacy of FDG PET/CT metabolic parameters

Metabolic parameters	Sensitivity	Specificity	cutoff value	AUC [95% CI]
SUV_max_	0.661	0.870	7.45	0.819 (0.743–0.895)
k_2_ (min ^− 1^)	0.669	0.739	0.338	0.729 (0.614–0.845)
k_3_ (min ^− 1^)	0.605	0.826	0.053	0.728 (0.631–0.826)
K_i_ (ml/g/min)	0.589	0.999	0.025	0.830 (0.761-0.900)

### Dynamic and static parameter analysis in malignant group

**Fig. 2:**
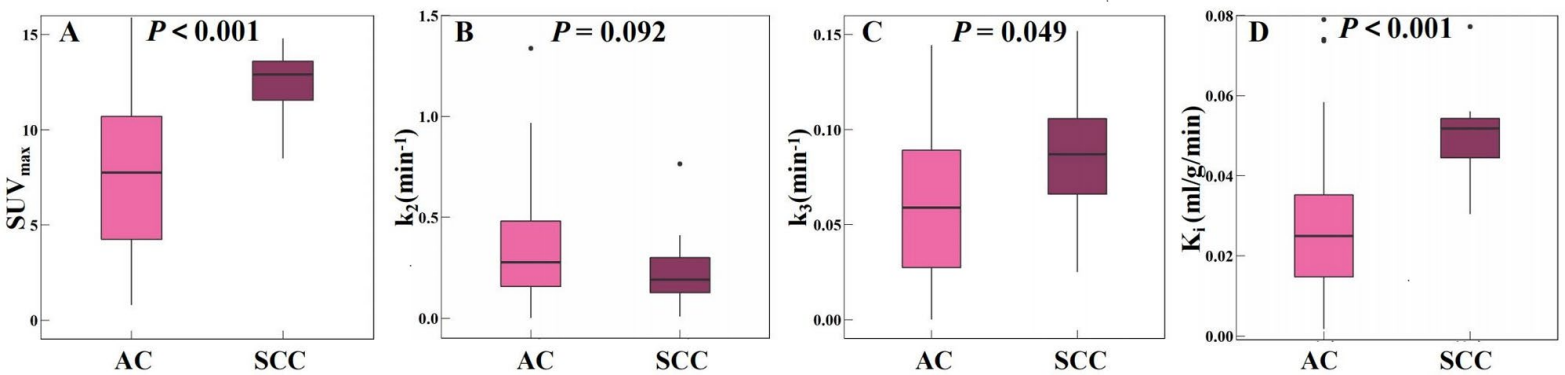
Parameter analysis of AC group and SCC group for both dPET/CT and sPET/CT

Based on the results of the previous section, we analyzed the metabolic parameters in the AC group and SCC group. Figure 2 shows the parameter analysis of the AC group and SCC group in both dPET/CT and sPET/CT.

In the AC group, the average SUV_max_ (Fig. 2A), k_3_ (Fig. 2C), and K_i_ (Figs. 2D and 7.95 [4.30;11.53], 0.0587 [0.0277;0.0888] min^− 1^ and 0.0247 [0.0145;0.0351] ml/g/min) were lower than SCC group (13.80 [12.70;16.40], 0.0798 [0.0537;0.0987] min^− 1^ and 0.0448 [0.0314;0.0534] ml/g/min), and the differences were all statistically significant (*P* < 0.001, *P* = 0.049, and *P* < 0.001, respectively). However, the k_2_ (Fig. 2B) did not show such a difference between AC and SCC groups (0.2828 [0.1626;0.5221] vs. 0.1987 [0.1263;0.3012] min^− 1^, *P* = 0.092).

### Dynamic and static parameter analysis in AC group

**Fig. 3:**
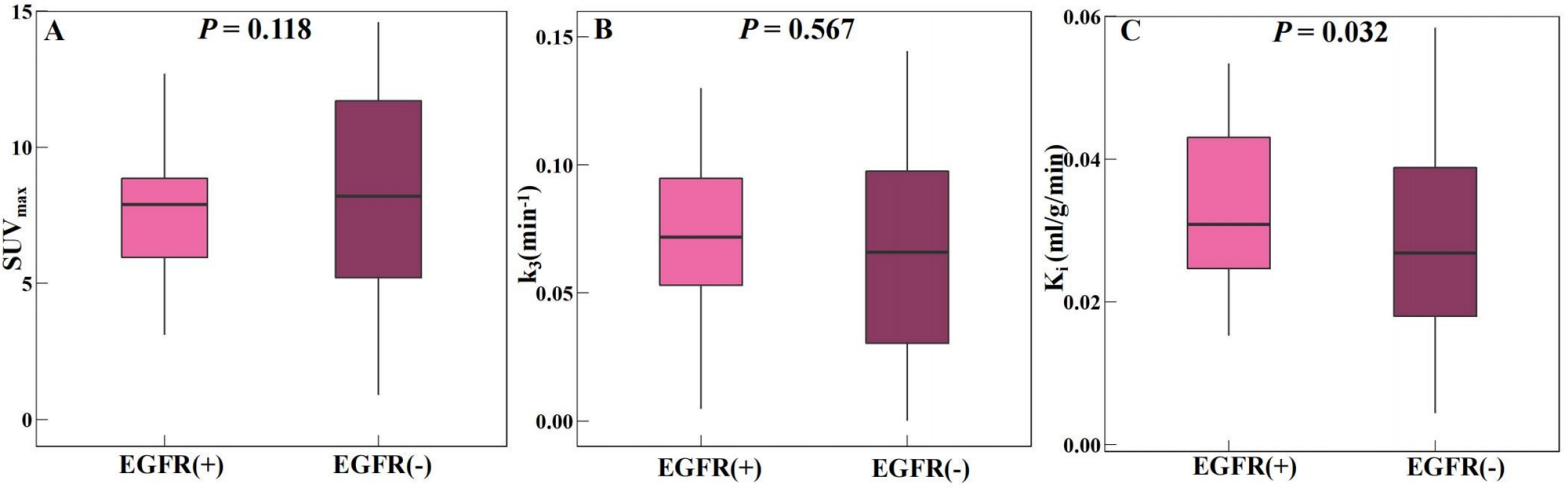
Parameter analysis of EGFR (+) and EGFR (-) groups for both dPET/CT and sPET/CT

Forty-eight patients with AC underwent EGFR status testing. Among them, 31 (64.58%) patients were in the EGFR (+) group, and 17 (35.42%) patients were in the EGFR (-) group. Figure 3showed the parameter analysis of the EGFR (+) group and EGFR (-) group in both dPET/CT and sPET/CT. In the EGFR (+) group, the average K_i_ (Fig. 3C, 0.0279 [± 0.0153] ml/g/min) was lower than EGFR (-) group (0.0405 [± 0.0200] ml/g/min), and the difference were statistically significant (*P* = 0.032). However, the SUV_max_ (Fig. 3A) and k_3_ (Fig. 3B) did not show such difference between EGFR (+) and EGFR (-) groups (9.28 [± 5.11] vs. 12.49 [± 7.25] and 0.0666 [± 0.0389] vs. 0.0730 [± 0.0354] min^− 1^, *P* = 0.118, *P* = 0.567, respectively).

### ROC analysis and cut-off values for K_i_ prediction of the EGFR mutation status

**Fig. 4:**
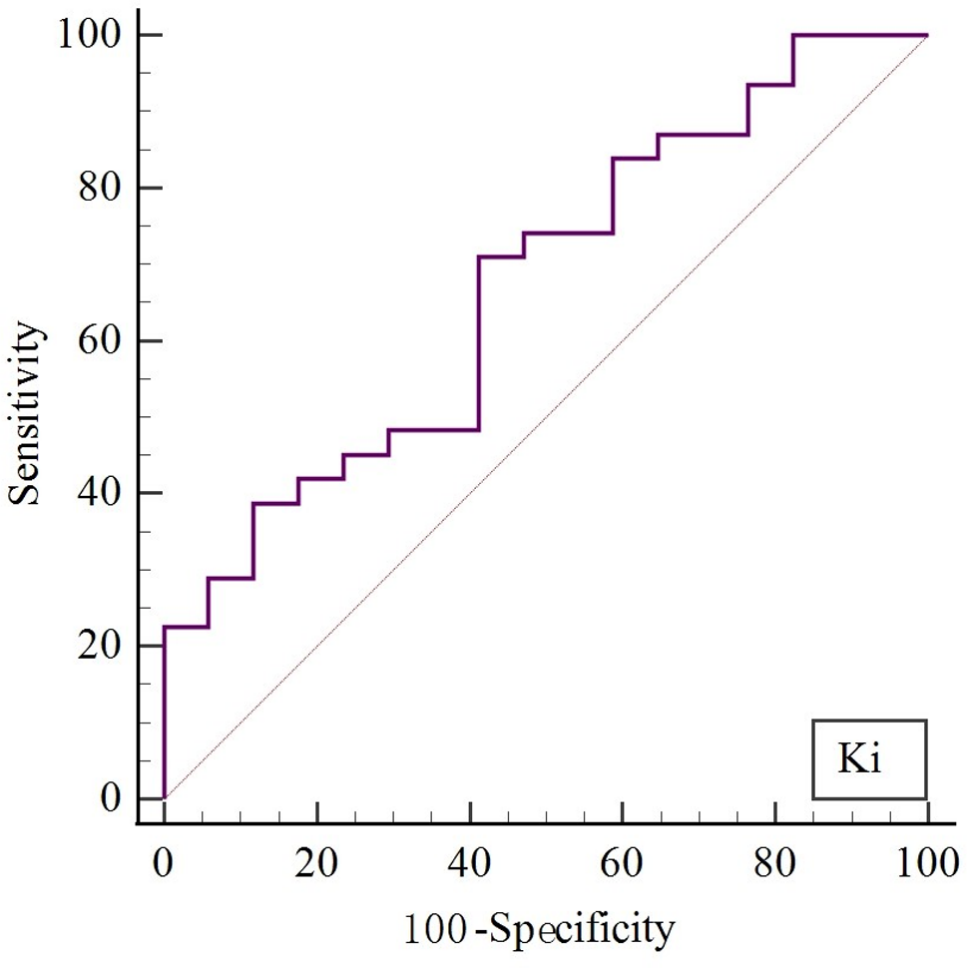
The ROC curves for the predicting EGFR mutation of K_i_

Based on the results of the previous section, we further performed ROC analysis to explore the capability of dynamic metabolic parameter K_i_ in predicting EGFR mutation status. For ROC analysis (Fig. 4), K_i_ had a cut-off value of 0.0350 ml/g/min when best predicting EGFR status, with a sensitivity of 0.710, a specificity of 0.588, and an AUC of 0.674 [0.523–0.802].

## Discussion

Clinical concerns have been raised about making the most of specific and accurate differential diagnoses of lung cancer to reduce the false-positive rate and develop individualized treatment plans. In this study, we found that both static metabolic parameters (SUV_max_), and dynamic metabolic parameters (K_i_) have good diagnostic value in the differential diagnosis of lung cancer. However, the specificity can be improved when the dynamic metabolic parameter K_i_ is added. Another finding was that among AC patients, K_i_ values were lower in EGFR (+) patients than in EGFR (-) patients, and for some patients with non-small cell lung cancer (NSCLC) where EGFR testing is not available, K_i_ improved its discriminability.

Since FDG is not a tumor-specific imaging agent, not only malignant tumors but also granulomatous diseases, concurrent infectious, and inflammatory diseases (tuberculosis, pneumonia, and interstitial lung disease) can exhibit FDG-avid [3–4]. As a result, uncertain PET signatures could lead to unnecessary biopsies or thoracotomies for some benign pulmonary lesions with high FDG metabolism. Deppen et al. [7] concluded that, in regions with endemic infectious lung disease, the specificity of FDG PET/CT for the differential diagnosis of lung cancer was overstated (specificity of 61% [49-72%]). In our study, 23 patients were confirmed as benign lesions (SUV range of 1.2–9.0) by surgical or puncture pathology results. Previously, Luo et al. used FDG PET/CT multi-time points imaging for differential diagnosis between AC and tuberculosis, but it has not been widely used in clinical practice [20]. Therefore, it is crucial to improve diagnostic specificity, thus allowing to operate early on malignant lesions and avoid unnecessary surgery in patients with benign lesions.

The compartmental model is regarded as the most accurate way to measure the uptake of FDG. Unlike static imaging, quantitative information on FDG metabolism was obtained through dynamic acquisition. By improving the description of the various stages of FDG metabolism, these metabolic parameters reflect the pathophysiological mechanisms. Huang et al. [21] concluded that in a small group of patients (*N* = 34), K_i_ can better identify benign and malignant solitary pulmonary nodules (0.004 vs. 0.023 ml/g/min, *P* = 0.0034) in areas (Taiwan) with a high prevalence of the granulomatous disease. Aleksander et al. [22] revealed that the lung malignancy group has higher K_i_ values than the benign group (0.0230 ± 0.0155 vs. 0.0057 ± 0.0071 ml/g/min) and could use it to better distinguish benign from malignant (*P* = 0.0311). Consistent with these researches, we found that both static metabolic parameters (SUV_max_) and dynamic metabolic parameters (including k_2_, k_3_, and K_i_) have good diagnostic value in the differential diagnosis of lung cancer. Parameter K_i_ was lower in the benign lesions than in the malignant lesions (0.0102 vs. 0.0267 ml/g/min, *P* < 0.001).

**Fig. 5:**
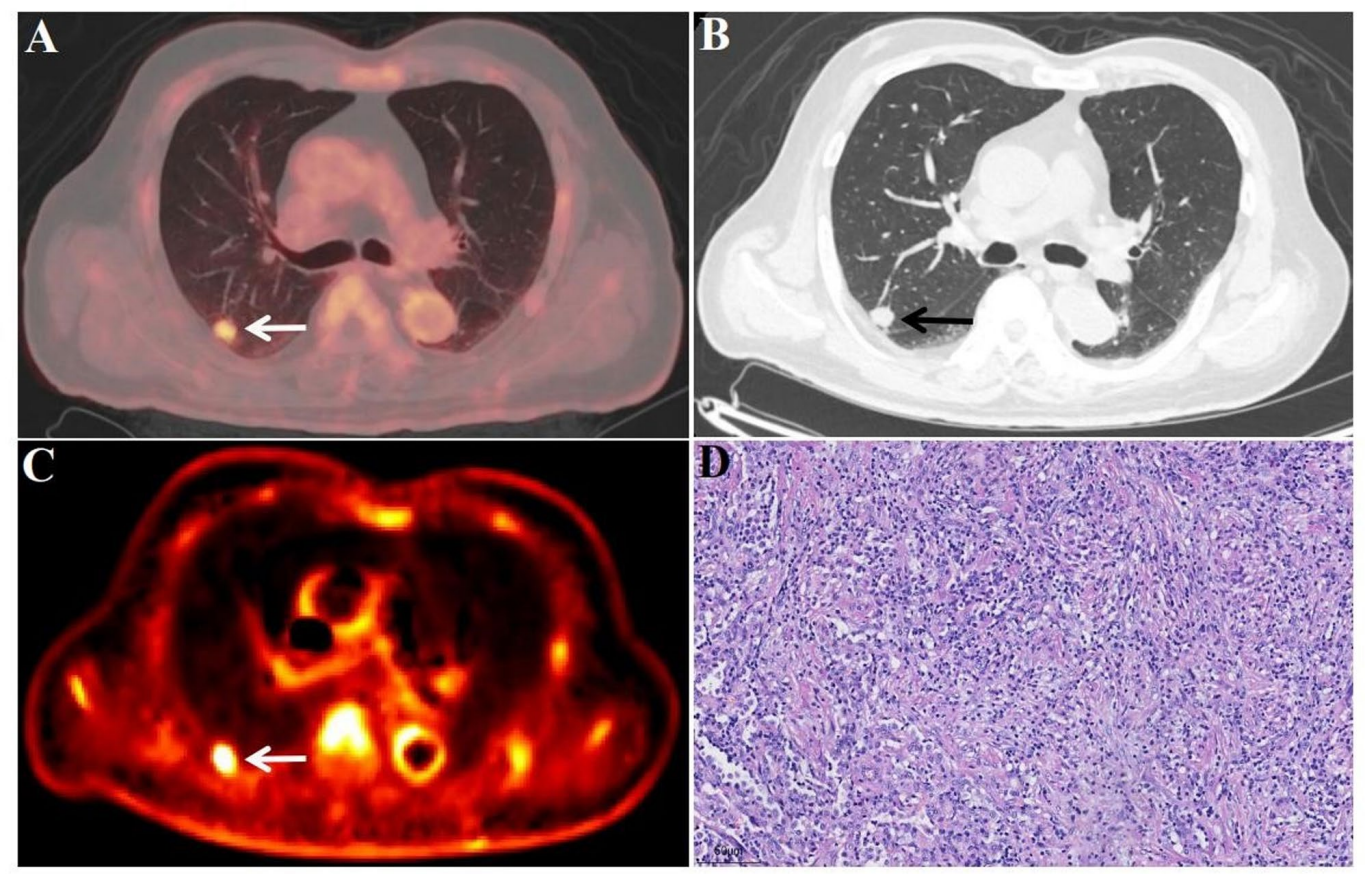
FDG-PET/CT images of a benign lesion A 66-year-old male patient. Surgical pathology confirmed an inflammatory lesion (D, 20x field of view) in the lower lobe of the right lung (white/black arrow), with a size of 1.4 × 1.2 cm (A and B, white/black arrow), SUV_max_ of 4.1 (A, white arrow), and K_i_ of 0.0102 ml/g/min (C, white arrow)

**Fig. 6:**
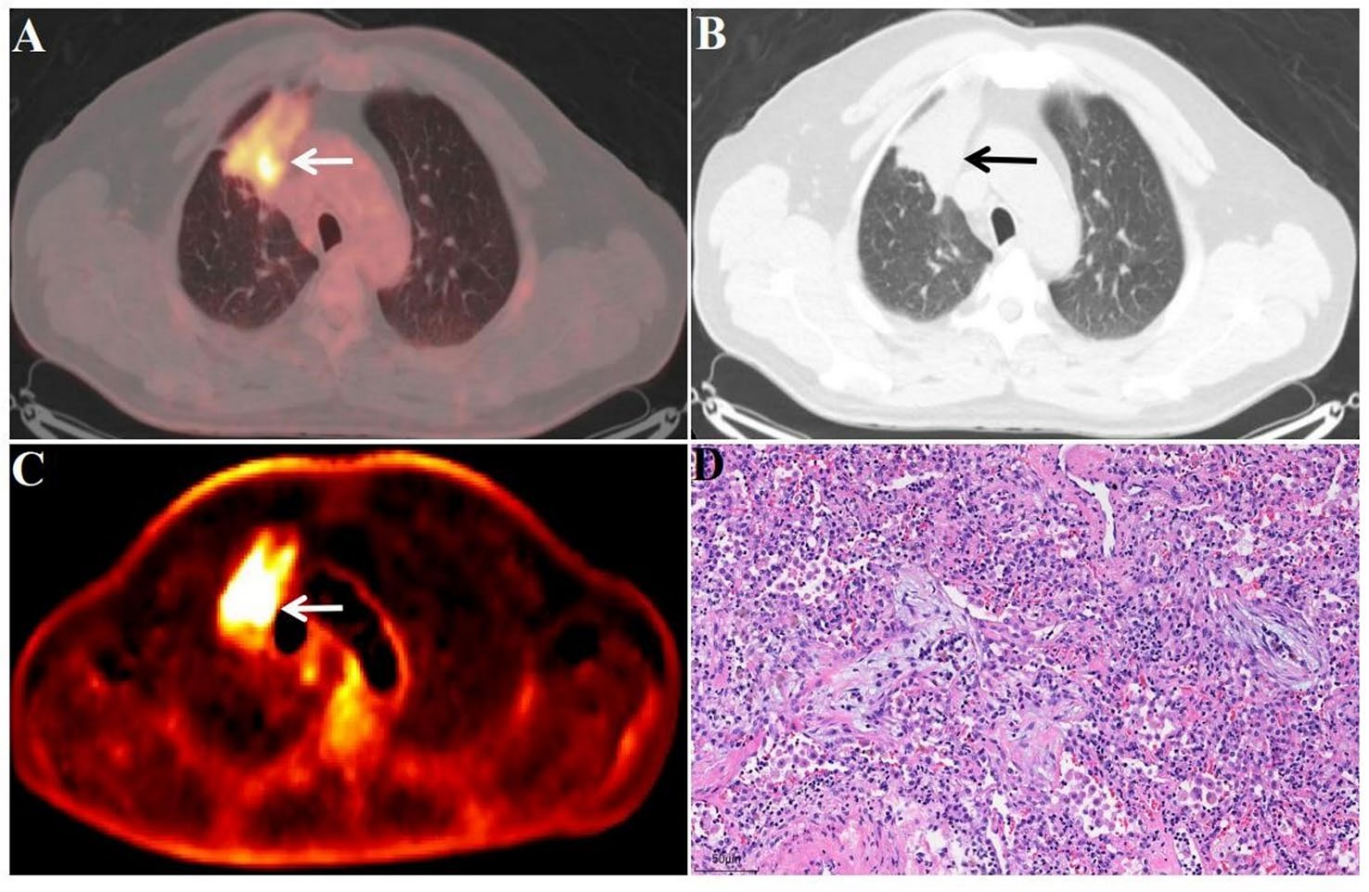
FDG-PET/CT images of a benign lesion A 59-year-old male patient. Surgical pathology confirmed an inflammatory lesion (D, 20x field of view) in the upper lobe of the right lung (white/black arrow), with a size of 5.3 × 4.5 cm (A and B, white/black arrow), SUV_max_ of 7.4 (A, white arrow), and K_i_ of 0.0120 ml/g/min (C, white arrow)

The ROC curve analysis revealed that both the static metabolic parameter SUV_max_ and the dynamic metabolic parameter K_i_ had good diagnostic values (AUC of 0.819 and 0.830). Compared with SUV_max_, the specificity of K_i_ has been further improved (0.870 vs. 0.999). In our study, 23 patients with SUV_max_ ranging from 1.2 to 9.0 had pathologically confirmed benign lesions after FDG PET/CT scan, while in contrast these patients had K_i_ values ranging from 0.0002 to 0.0246 ml/g/min (Figs. 5C and 6C). Therefore, the specificity of the differential diagnosis can be improved when the cut-off value of K_i_ was 0.0250 ml/g/min, especially for patients with FDG-avid lesions. This may reduce unnecessary invasive tests/treatments.

The previous study concluded that, in lung cancer, SUV_max_ and K_i_ values of AC were lower than those of SCC (9.14 ± 1.48 vs. 5.58 ± 0.62 and 0.052 ± 0.009 vs. 0.029 ± 0.004 min^− 1^, *P*<0.05) [23]. Tineke et al. concluded that AC had lower k_3_ values than SCC in lung cancer [24]. In this study, we found that the SUV_max_, k_3_, and K_i_ values in the AC group were lower than those in the SCC group, similar to previous reports.

EGFR can mediate oncogenic signals involved in the proliferation and survival of tumor cells and is expressed and activated in a variety of epithelial malignancies [25]. EGFR status has become a major prognosis factor. Previous studies have shown that treatment of patients with EGFR activating and sensitizing mutation-driven NSCLC with EGFR tyrosine kinase inhibitors (TKIs) achieved a response rate (RR) of 60–80% with a median progression-free survival (PFS) of 8–13 months [26–28]. Improved quality of life in EGFR (+) patients treated with gefitinib can be achieved when compared with standard chemotherapy [27–29]. In clinical practice, EGFR testing is not available for some patients since high-quality genetic testing of tumor tissue is challenging due to many factors. Therefore, it is crucial to identify reliable metabolic parameters for non-invasive prediction of EGFR status based on FDG PET/CT imaging.

**Fig. 7:**
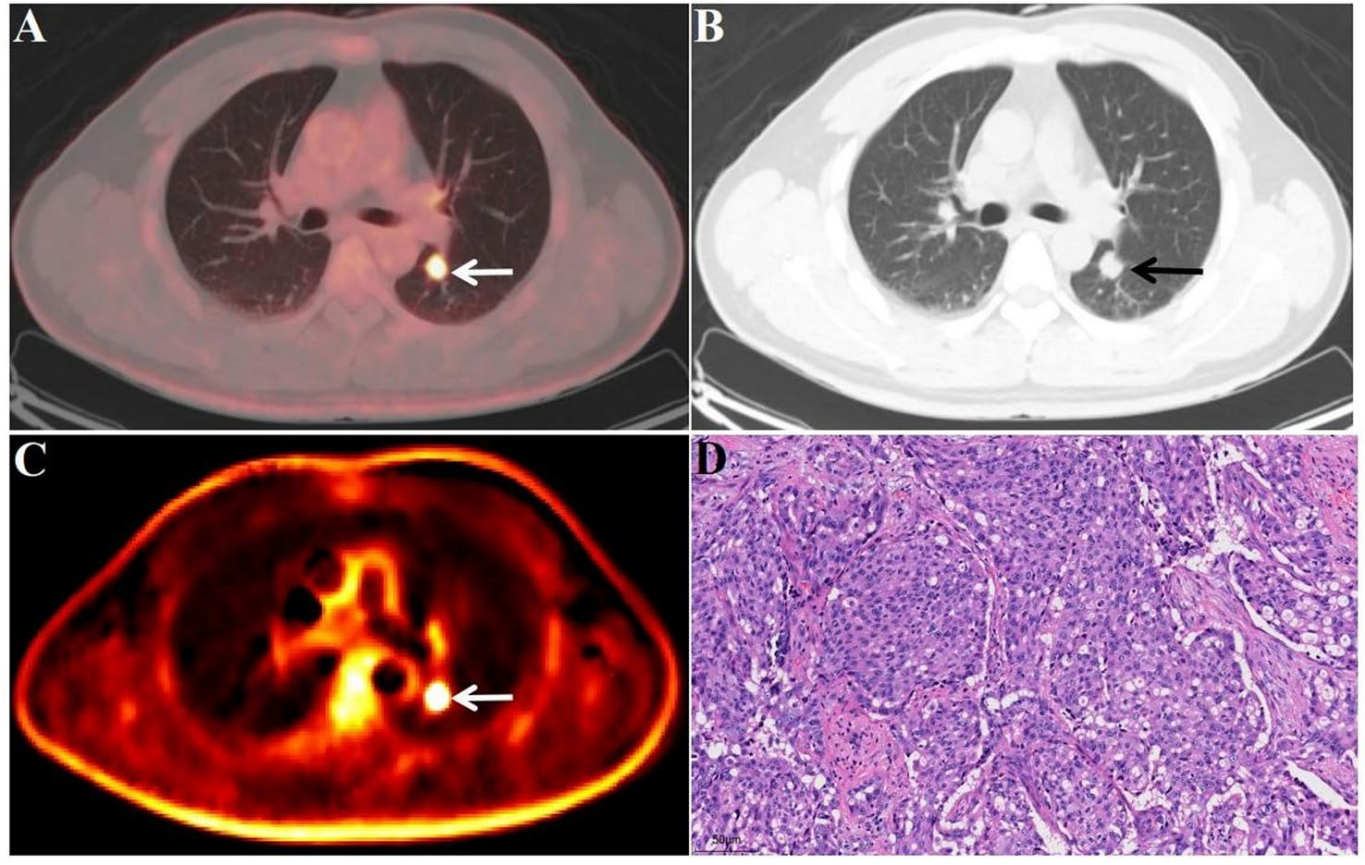
FDG-PET/CT images of a malignant lesion A 41-year-old male patient. Surgical pathology confirmed an adenocarcinoma (D, 20x field of view) in the upper lobe of the left lung (white/black arrow), with a size of 1.6 × 1.5 cm (A and B, white/black arrow), SUV_max_ of 13.8 (A, white arrow), and K_i_ of 0.0282 ml/g/min (C, white arrow). Postoperative EGFR test results showed EGFR exon 19 mutation

**Fig. 8:**
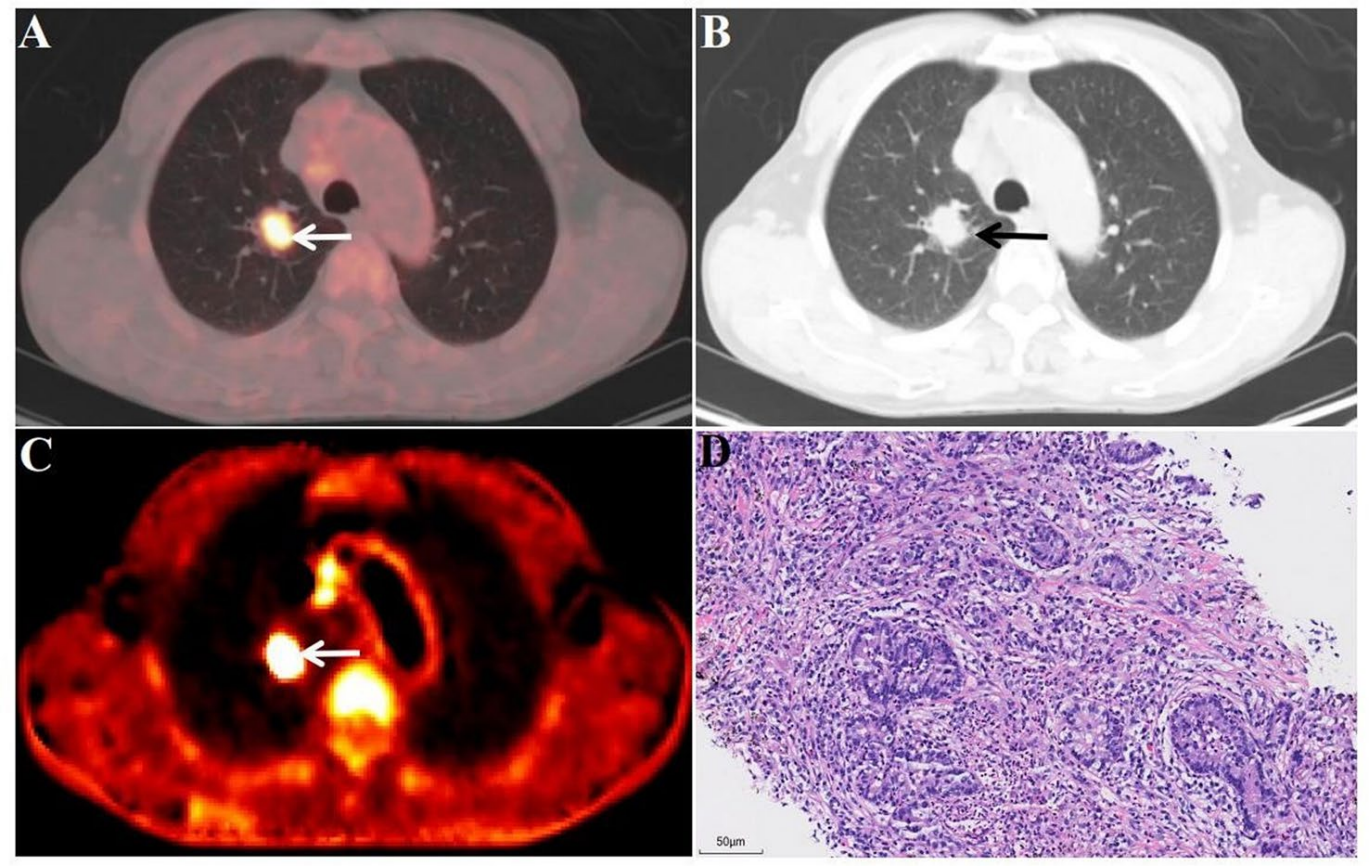
FDG-PET/CT images of a malignant lesion A 59-year-old male patient. Surgical pathology confirmed an adenocarcinoma (D, 20x field of view) in the lower lobe of the right lung (white/black arrow), with a size of 2.0 × 1.6 cm (A and B, white/black arrow), SUV_max_ of 7.9 (A, white arrow), and K_i_ of 0.0442 ml/g/min (C, white arrow). Postoperative EGFR testing was negative

Numerous prior studies have been conducted regarding the prediction of EGFR status based on SUV_max_, however, the results have not been satisfactory. Huang et al. [30] concluded that higher SUV_max_ values in lung adenocarcinoma patients are more likely to develop EGFR mutations. Subsequently, it has also been concluded that low SUVmax values are associated with EGFR mutations in patients with NSCLC [31, 32]. Carlos Caicedo et al. [33] concluded that the presence of EGFR mutations was not correlated with FDG uptake. In our study, in the AC group, the SUV_max_ did not show difference between EGFR (+) and EGFR (-) groups. However, we found that K_i_ values were lower in the EGFR (+) group than in the EGFR (-) group (0.0279 vs. 0.0405 ml/g/min) with statistically significance (*P* = 0.032). For ROC analysis, K_i_ had a cut-off value of 0.0350 ml/g/min for predicting EGFR status, with a sensitivity of 0.710, a specificity of 0.588, and an AUC of 0.674 (Figs. 7C and 8C). Therefore, the including of the dynamic metabolic parameter K_i_ provides more metabolic information and is expected to be a means of non-invasive de-prediction of the status of EGFR. In particular, patients who are unable or unavailable for EGFR testing are likely to benefit.

Our study has several limitations. First, in this study, we have a small percentage of patients in the benign and SC groups, so the main results have focused on the AC group. In the future, we will expand the sample size to continue related studies for all groups. Second, motion correction was not considered in this study. It is known that motion in the chest region can affect not only the SUV but also the kinetic parameters quantification [34–37]. Dedicated quality control and motion correction process may be required to obtain accurate quantification before proceeding with the evaluation. Third, SUV_max_ rather than SUV_mean_ was used in this study as we considered SUV_max_ was less affected by the partial volume effects [38–40]. Last, only imaging features were applied for diagnosis. A future direction would be to see if adding clinical factors into the image-based features, i.e. as a multivariable model, could provide additional values in differential diagnosis.

## Conclusion

Both static metabolic parameters (SUV_max_) and dynamic metabolic parameters (k_2_, k_3_, and K_i_) have good value in the differential diagnosis of lung cancer. When the cut-off value of K_i_ is 0.0250 ml/g/min, the specificity of the differential diagnosis of lung cancer can be improved. When the cut-off value of K_i_ was 0.0350 ml/g/min, the sensitivity for predicting EGFR status was 0.710. For patients for whom EGFR testing is not available, dynamic imaging may become an important non-invasive screening tool.

## Data Availability

The datasets of the current study are available from the corresponding author on reasonable request.
